# Selection of the Amino Acid and Saccharide That Increase the Tetracycline Susceptibility of *Vibrio splendidus*

**DOI:** 10.3389/fvets.2021.823332

**Published:** 2022-01-28

**Authors:** Guohua Jiang, Yanan Li, Ya Li, Weiwei Zhang, Chenghua Li

**Affiliations:** ^1^Collaborative Innovation Center for Zhejiang Marine High-efficiency and Healthy Aquaculture, Ningbo University, Ningbo, China; ^2^State Key Laboratory for Quality and Safety of Agro-products, Ningbo University, Ningbo, China; ^3^Laboratory for Marine Fisheries Science and Food Production Processes, Qingdao National Laboratory for Marine Science and Technology, Qingdao, China

**Keywords:** *Vibrio splendidus*, persister cells, carbon source, chemotaxis, antibiotic susceptibility

## Abstract

Bacterial persister cells are a subpopulation of isogenic bacteria with characteristics of reduced metabolic activity and multidrug antibiotic resistance. Our lab had previously proved that *Vibrio splendidus* could form persister cells both naturally and after stimulation. However, the conditions for the waking up of *V. splendidus* persister cells remain marginal. In this study, the carbon sources that could wake up *V. splendidus* persister cells were selected from 20 amino acids and eight saccharides. The result showed that L-glutamic acid, L-aspartic acid, L-arginine, L-phenylalanine, L-leucine, maltose, D-galactose, sorbitol, mannose, N-acetyl-D-glucosamine, D-glucose, and D-fructose could wake up the *V. splendidus* persister cells. The chemotaxis activity of both exponential cells and regrown persister cells on plate containing each of the selected carbon source are also high. The existence of the selected carbon source can affect the antibiotic susceptibility of *V. splendidus*. When L-glutamic acid, L-aspartic acid, L-phenylalanine, and D-glucose were separately added into the cultured *V. splendidus* simultaneously with tetracycline, *V. splendidus* could be completely eliminated, while the addition of L-alanine and D-galactose could not. Our study suggested that *V. splendidus* persister cells could revive in the presence of specific carbon sources, and the addition of these exogenous nutrients could increase the tetracycline susceptibility of *V. splendidus*.

## Introduction

Persister cells are a little potion of bacterial cells which are temporarily resistant to multidrug on account of a metabolic transient alteration (1). In the 1940s, it was found that some bacteria were not killed completely after treatment with antibiotics (2). In 1944, Bigger named these surviving subpopulation “persister cells” (3). Both groups determine that persister cells are dormant, and this conclusion has been corroborated from that time on (4, 5). Unlike resistant cells, the genes of the persister cells did not alter (6, 7), but they are a little potion of bacterial cells with a transient state of hypometabolic or dormancy that can help bacteria to survive the exterior stresses, such as antibiotic treatment and nutrient deficiency (8–11). Till now, this phenotype has been commonly found in *Staphylococcus aureus, Pseudomonas aeruginosa, Escherichia coli*, Vibrio splendidus and Archaea (12–14) during *in vitro* growth as well as infection in the host.

Up to now, more and more studies have shown that persister cells play an important part in the resuscitation of infection diseases. Persister cells avoid antibiotic killing by reducing metabolism, and when the level of antibiotics in the external environment drops, the persister cells resume growing (3, 12, 15, 16). Therefore, the persister cells lead to low efficacy of antibiotic treatment and high occurance of repeated infection. Different kinds of persister cells use specific carbon sources to wake up. For example, the persister cells of *P. aeruginosa* resuscitated in the condition of L-proline (17), while the persister cells of *E. coli* revived in the presence of L-alanine (18). Furthermore, the carbon sources that wake up the persister cells affect the antibiotic susceptibility of persister cells. The changes in antibiotic susceptibility triggered by glucose are documented in *E. coli, Vibrio cholerae, P. aeruginosa*, and *S. aureus* (19–22). The antibiotic sensitivity of *E. coli, S. aureus, Edwardsiella tarda, Edwardsiella piscicida*, and *P. aeruginosa* are also affected by fructose, maltose, sucrose, leucine, glycine, and alanine (19–21, 23–25).

*V. splendidus* is a gram-negative bacterium that is ubiquitously presented in marine ecosystems (26), and it is a significant opportunistic bacterial pathogen which causes infection of marine shellfish and sea cucumber *Apostichopus japonicus* (27–30). Our previous study showed that *V. splendidus* could form persister cells (14). To further know more about *V. splendidus* persister cells, the carbon sources that could wake up *V. splendidus* persister cells were selected in this study, and furthermore, a strategy for completely eliminating *V. splendidus* was proposed.

## Materials

### Bacterial Strains and Culture Conditions

*V. splendidus* was cultured in 2216E medium at 28°C (1 g yeast extract; 5 g tryptone; and 0.01 g FePO_4_, 1 L seawater). M9 minimal medium was prepared as follows: 10 ml 0.1 M CaCl_2_, 10 ml 0.1 M MgSO_4_, 840 ml ddH_2_O, 100 ml sterilized 10 × salt (20 g NaCl, 30 g KH_2_PO_4_, 70 g Na_2_HPO_4_, 10 g NH_4_Cl in 1 L ddH_2_O). The concentration of each saccharide was 0.4%, which was used to wake up *V. splendidus* persister cells. The amino acids and their corresponding levels used to wake up *V. splendidus* persister cells are listed in Table 1 (31). Tetracycline was dissolved in pure ethanol to make a stock solution of 10 mg·ml^−1^.

**Table 1:** Levels (5 × concentration) of amino acids in M9 minimal medium.

**Amino acid**	**Final concentration (μg/ml)**	**Stock**	**Amount per liter**
L-alanine	75	1%	7.5 ml
L-arginine	145	2%	7.25 ml
L-asparagine	75	1%	7.5 ml
L-aspartic acid	75	1%	7.5 ml
L-cysteine	50	2%	2.5 ml
L-histidine	42	2%	2.1 ml
L-glutamic acid	75	1%	7.5 ml
L-glutamine	75	1%	7.5 ml
L-glycine	110	2%	5.5 ml
L-isoleucine	42	1%	4.2 ml
L-leucine	41	1%	4.1 ml
L-lysine	75	1%	7.5 ml
L-methionine	25	2%	1.25 ml
L-phenylalanine	75	1%	7.5 ml
L-proline	164	4%	4.1 ml
L-serine	42	2%	2.1 ml
L-threonine	82	2%	4.1 ml
L-tryptophan	18	0.25%	7.1 ml
L-tyrosine	75	1%	7.5 ml
L-valine	42	1%	4.2 ml

### Selection of Persister Cells

*V. splendidus* persister cells were prepared by lysing active cells with high concentration of tetracycline 400 μg·ml^−1^, according to our previous study (14). Briefly, 10 ml of overnight *V. splendidus* culture was mixed with 40 ml 2216E containing 2,000 μl of tetracycline stock solution, and the culture was incubated 4 h in a shaker with a shaking speed of 150 rpm. The remaining bacteria were washed with 2% NaCl three times. To verify whether these remaining cells were persister cells, we observed their status on agarose gel pads without any carbon sources as described by Kim et al. (32). In short, agarose was put into 44 ml ddH_2_O at 1.5% and sterilized by autoclaving; then, 5 ml 10 × M9 salt solution, 0.5 ml 0.01 M CaCl_2_, and 0.5 ml 0.1 M MgSO_4_ solution were subsequently added before the gel was solidified. The cells were observed under the optical microscope (ZEISS) maintained at 28°C.

### *V. splendidus* Persister Cells Revived on Amino Acids

The selection of amino acid for the resuscitation of *V. splendidus* persister cells was determined as reported previously (17). Twenty amino acids were divided into four combinations, and five amino acids were in one group; #1: Phe, Ala, Cys, Ser, and Arg, #2: Tyr, Val, Gly, Thr, and His, #3: Trp, Ile, Pro, Asn, and Lys, and #4: Met, Leu, Gln, Glu, and Asp. To explore the recovery of persister cells on a single amino acid, we separately tested 15 amino acids in combination #1, #3, and #4. *V. splendidus* persister cells were 10-fold serially diluted, and 100 μl of diluent were spread onto M9 minimal plates containing each kind of amino acid as carbon source, and the plates were incubated at 28°C for 96 h.

### *V. splendidus* Persister Cells Revived on Saccharides

Persister cells were also woken up by recognizing saccharides (18). To explore whether saccharides could be used as carbon source to resuscitate *V. splendidus* persister cells, eight saccharides, including maltose, D-glucose, D-ribose, mannose, D-galactose, sorbitol, N-acetyl-D-glucosamine and D-fructose, were separately added into M9 minimal medium. These saccharides are chosen because they are usually good carbon sources for bacteria, especially *N*-acetyl-D-glucosamine which is used as principal source of carbon and nitrogen by marine bacteria (33). *V. splendidus* persister cells were 10-fold serially diluted, and 100 μl of diluent were spread onto M9 minimal plates containing each kind of saccharide as carbon source, and the plates were incubated at 28°C for 48 h.

### Chemotaxis Assays

The chemotaxis of both exponential cells and regrown persister cells were performed as reported previously by Weng et al. (34). Briefly, *V. splendidus* were cultured until an optical density at 600 nm (OD_600_) of 1.0 and washed twice with M9 minimal medium. *V. splendidus* persister cells were obtained as previously described, and the persister cells were put into 1 ml M9 minimal medium. Amino acid or saccharide solutions were separately added onto M9 minimal plates containing 0.2% (w/v) agar. Ten microliter of bacterial suspensions were dropped on the M9 minimal plates containing each kind of individual carbon source, respectively, and the plates were incubated at 28°C for 96 h.

### Antibiotic Susceptibility

The overnight grown stationary phase culture was supplemented with each exogenous carbon source at levels of 0, 1, 5, 10, 15, 20, 30, and 40 mM, respectively, and tetracycline stock solution was added into the medium at a concentration of 400 μg·ml^−1^ immediately. The culture without addition of any exogenous carbon sources was used as a control. All the cultures were incubated at 28°C for 6 h with shaking at 150 rpm. After treatment, the cells were collected by centrifugation, diluted in 10-fold serially, and 10 μl of each diluent was dropped on 2216E plates. After incubation at 28°C for 24 h, the colonies that emerged on the plates were counted. The relative percent of survival was determined as follows: the cell number after tetracycline plus exogenous carbon source challenge/the cell number after tetracycline solo challenge.

### Strain Number and Statistical Analysis

The isolates of *V. splendidus* were deposited into the China General Microbiological Culture Collection (CGMCC, Beijing, China) with strain No. 7.242. Statistical analyses were performed by using the two tailed *t*-test. Statistical significance was determined by one-way ANOVA. In all cases, the significance level was defined as ^*^p < 0.05 and ^**^p < 0.01.

## Results

### Tetracycline-Selected *V. splendidus* Persister Cells

**Figure 1:**
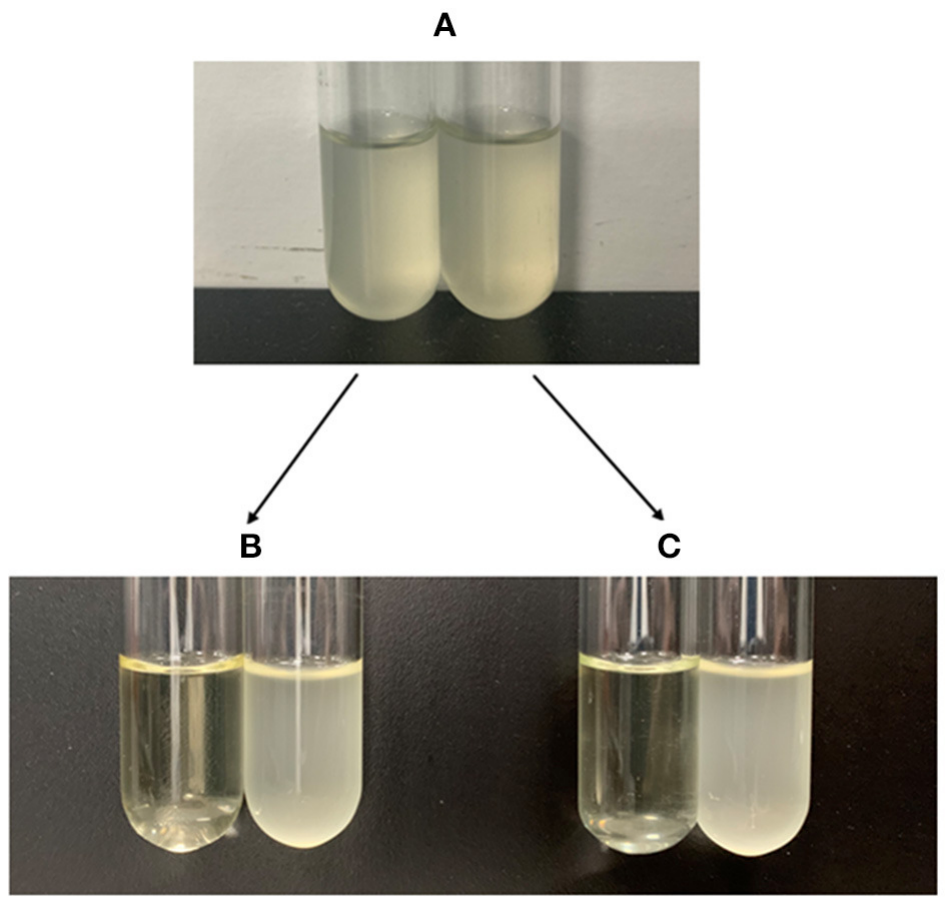
The antibiotic susceptibility of the tetracycline-selected *V. splendidus* persister cells and *V. splendidus* cells. **(A)** The growth of *V. splendidus* exponential cells (left) and regrowth of *V. splendidus* persister cells (right); **(B)** the exponential cells of *V. splendidus* with (left) and without (right) 40 μg·ml^−1^ tetracycline treatment; **(C)** the regrown cells of *V. splendidus* persister cells with (left) and without (right) 40 μg·ml^−1^ tetracycline treatment.

**Figure 2:**
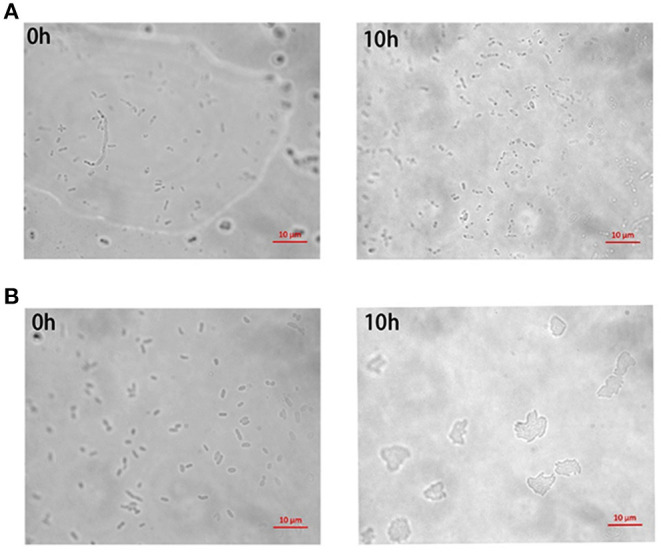
The division of *V. splendidus* persister cells and *V. splendidus* exponential cells in the lack of nutrition. **(A)** Division of *V. splendidus* persister cells on M9 minimal gel pads that without nutrition at the time points of 0 h and 10 h, respectively. **(B)** Division of *V. splendidus* exponential cells at time points of 0 h and 10 h in the lack of nutrition. Scale bar indicates 10 μm.

A portion of antibiotic-resistant *V. splendidus* cells emerged after 400 μg·ml^−1^ tetracycline was treated for 4 h at 28°C. These cells were confirmed as persister cells by measuring their antibiotic sensitivity and cell division. The result showed that the regrown cells from persister cells had equal susceptibility to the tetracycline, and 40 μg·ml^−1^ tetracycline could completely lyse both the regrown cells from persister cells and the exponential cells (Figure 1). The division of cells in the absence of nutrients was observed under microscope. It showed that the persister cells did not resuscitate within 10 h on agarose gel pads without nutrients (Figure 2A), while exponential cells of *V. splendidus* showed several divisions on agarose gel pads (Figure 2B). All these results showed that the cells obtained after treatment with high concentration of tetracycline were persister cells. In addition, the persister cells in the stationary culture of *V. splendidus* were determined to be at a potion of approximately 0.1%-1% determined by the colony that emerged on the plates.

### *V. splendidus* Persister Cells Resuscitated on Specific Amino Acid

**Figure 3:**
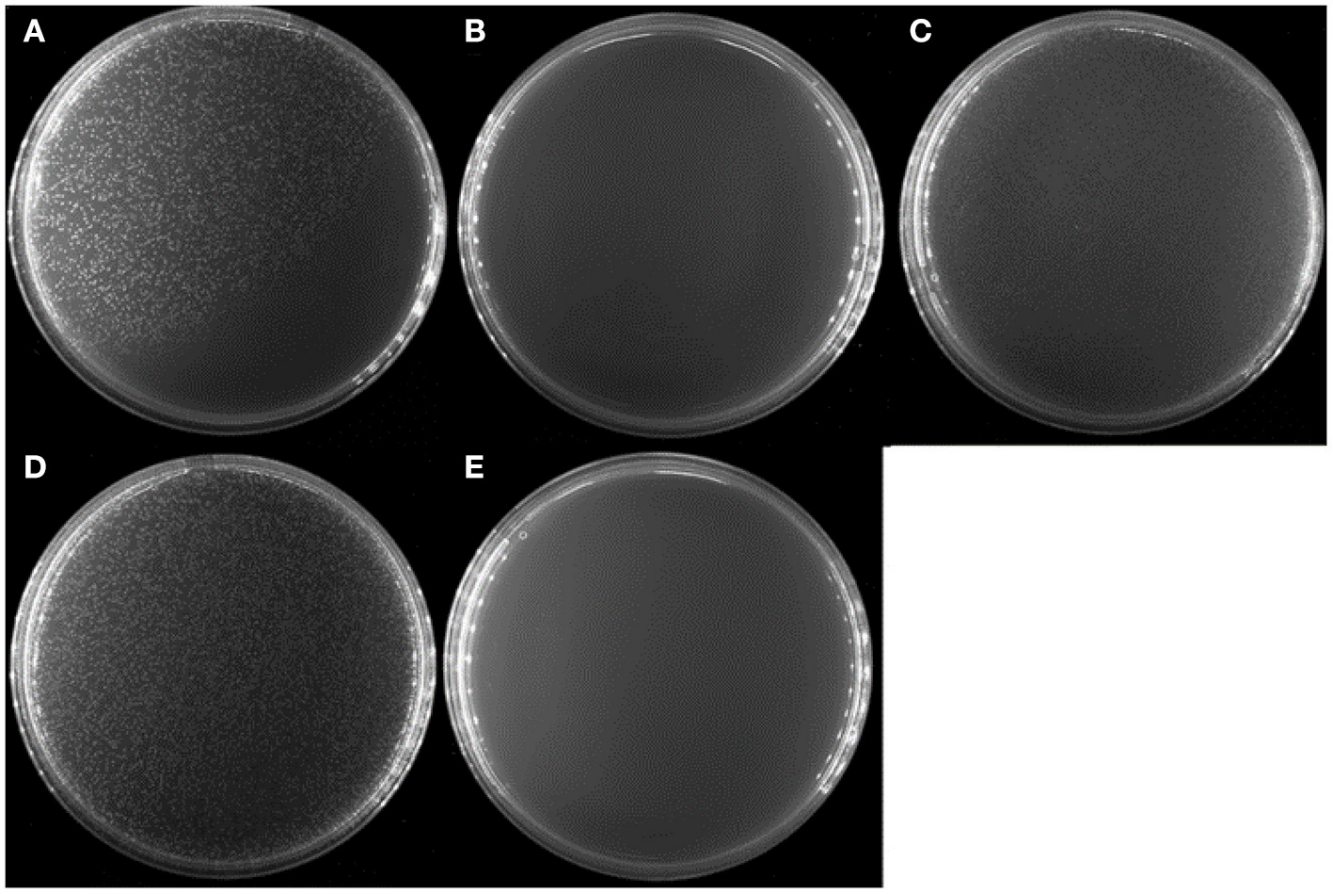
Persister cells wake up on amino acids combination plate agar. *V. splendidus* persister cells were incubated at 28°C for 96 h on M9 minimal plate agar with four different amino acid combinations, respectively: **(A)** #1: Phe, Ala, Cys, Ser, and Arg; **(B)** #2: Tyr, Val, Gly, Thr, and His; **(C)** #3: Trp, Ile, Pro, Asn, and Lys; **(D)** #4: Met, Leu, Gln, Glu, and Asp; **(E)** without amino acid. All amino acids in the experiment were 5 × concentration as listed in Table 1.

**Figure 4:**
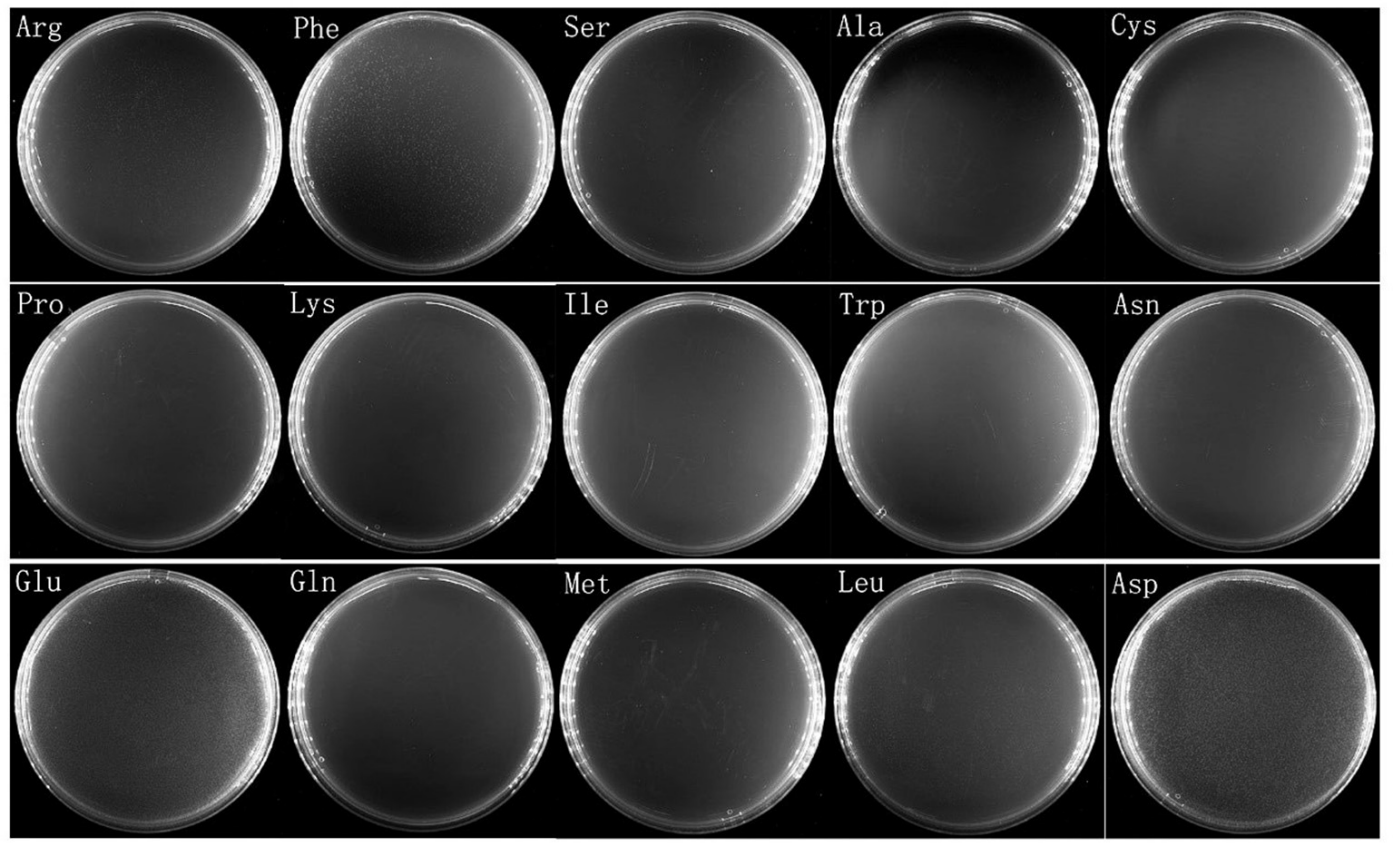
Persister cells wake up on plate containing single amino acid. *V. splendidus* persister cells were incubated at 28°C for 96 h on M9 minimal plate with 15 individual amino acids (L-alanine, L-serine, L-cystine, L-arginine, L-lysine, L-phenylalanine, L-asparagine, L-proline, L-isoleucine, L-tryptophan, L-glutamine, L-leucine, L-aspartic acid, L-methionine, and L-glutamic acid). The concentrations of the amino acids in the experiment were 5 × concentration as listed in Table 1.

To see whether *V. splendidus* persister cells could resuscitate on specific carbon source like the spores of *Bacillus subtilis*, a total of 20 amino acids were separately used as carbon source to wake up *V. splendidus* persister cells. For the first assay, we divided 20 amino acids into four groups as four kinds of carbon sources. The result showed that *V. splendidus* persister cells did not resuscitate on combination #2, but they could resuscitate on combinations #1, #3, and #4 (Figure 3). The 15 amino acids in combinations #1, #3, and #4 were further chosen to detect the single amino acid to wake up *V. splendidus* persister cells. Result showed that *V. splendidus* persister cells woke up at 17 h when L-glutamic acid was used as the only carbon source, which was the fastest among all the 20 amino acids (Figure 4). *V. splendidus* persister cells revived on M9 minimal plate with L-aspartic acid at 24 h, and they revived on M9 minimal plates with solo L-leucine, L-phenylalanine, and L-arginine when the resuscitation time increased to 40 h (Figure 4).

### *V. splendidus* Persister Cells Resuscitated on Specific Saccharide

**Figure 5:**
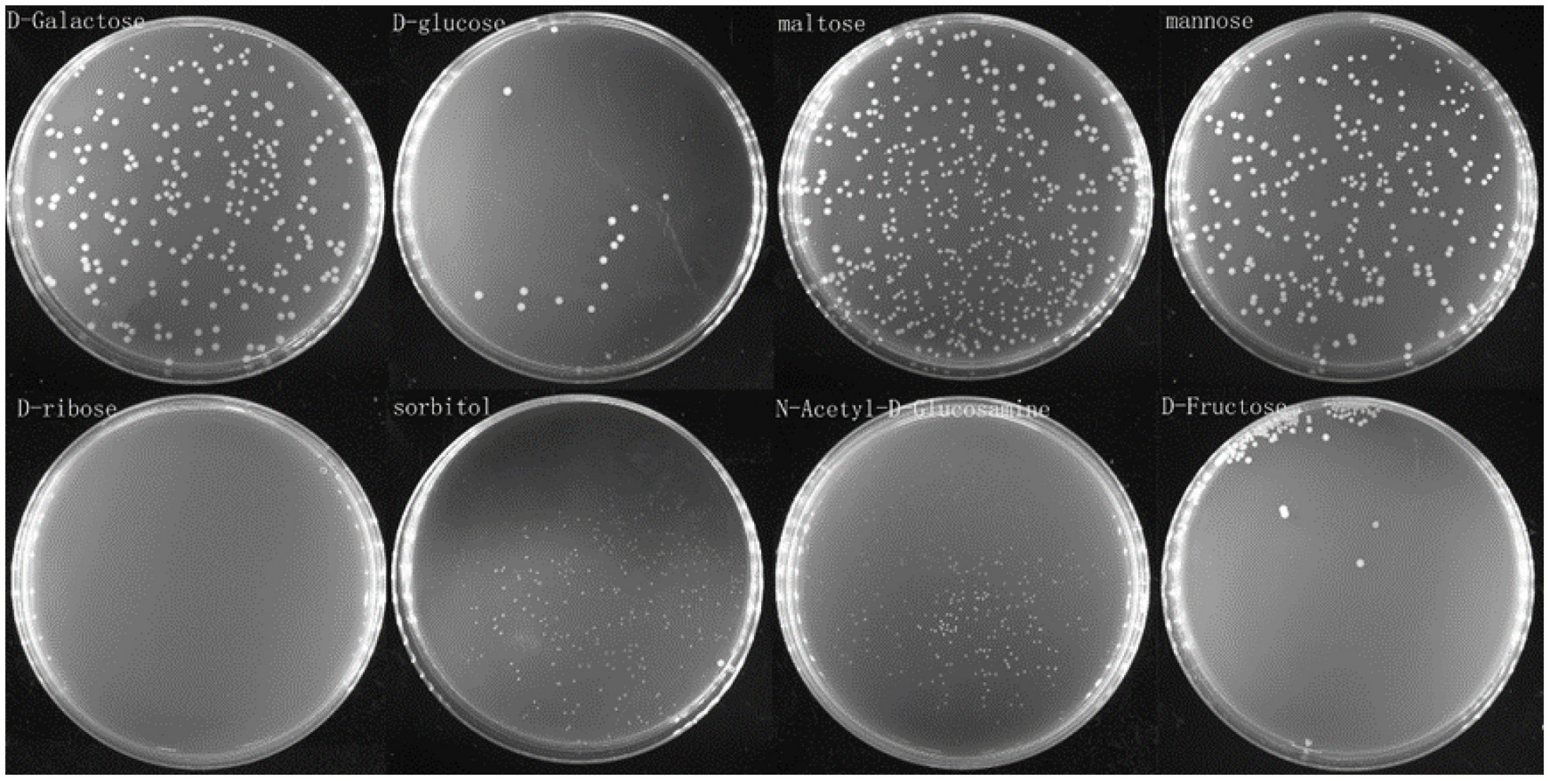
Persister cells wake up on plate containing single saccharide. Persister cells were spread on M9 minimal plates with D-glucose, D-fructose, D-ribose, N-acetyl-D-glucosamine, D-galactose, mannose, maltose, and sorbitol, respectively. The concentration of each saccharide on the plate was 0.4%, and the plates were incubated at 28°C for 48 h.

To see whether *V. splendidus* persister cells could resuscitate on saccharides, eight different kinds of saccharides were separately used as carbon source for *V. splendidus* persister cells to revive. Our result showed that *V. splendidus* persister cells revived on M9 minimal plate supplemented with D-galactose and maltose at 24 h and they revived on mannose and sorbitol at 36 h, but *V. splendidus* persister cells slowly revived on M9 agar plate with separate D-fructose, N-acetyl-D-glucosamine, and D-glucose when the resuscitation time increased to 48 h. Less colony numbers were obtained when the persister cells were revived on D-fructose and D-glucose, but larger colonies were obtained when they were revived on D-fructose and D-glucose compared with N-acetyl-D-glucosamine. However, *V. splendidus* persister cells did not revive with D-ribose at all within 48 h (Figure 5).

### *V. splendidus* Showed Chemotaxis in Different Carbon Sources

**Figure 6:**
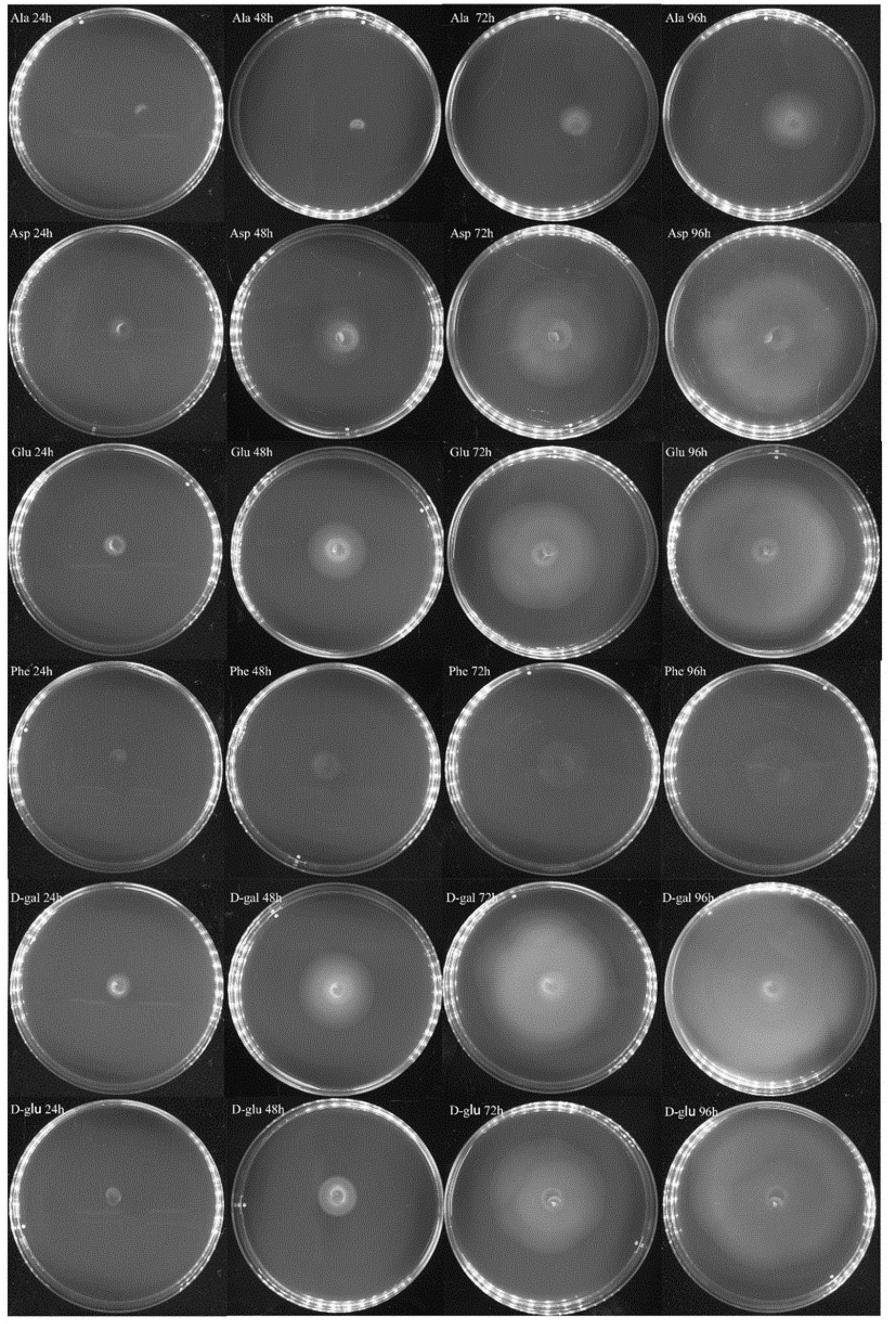
Chemotaxis of *V. splendidus* exponential cells. Each exogenous carbon source at a concentration of 400 mg·L^−1^ was separately added into M9 minimal plates containing 0.20% (w/v) agar; 1 ml M9 minimal medium was used to resuspend persister cells, and 10 μl cell suspensions was dropped on the M9 minimal plate containing each carbon source, and the plates were incubated at 28°C for 96 h.

**Figure 7:**
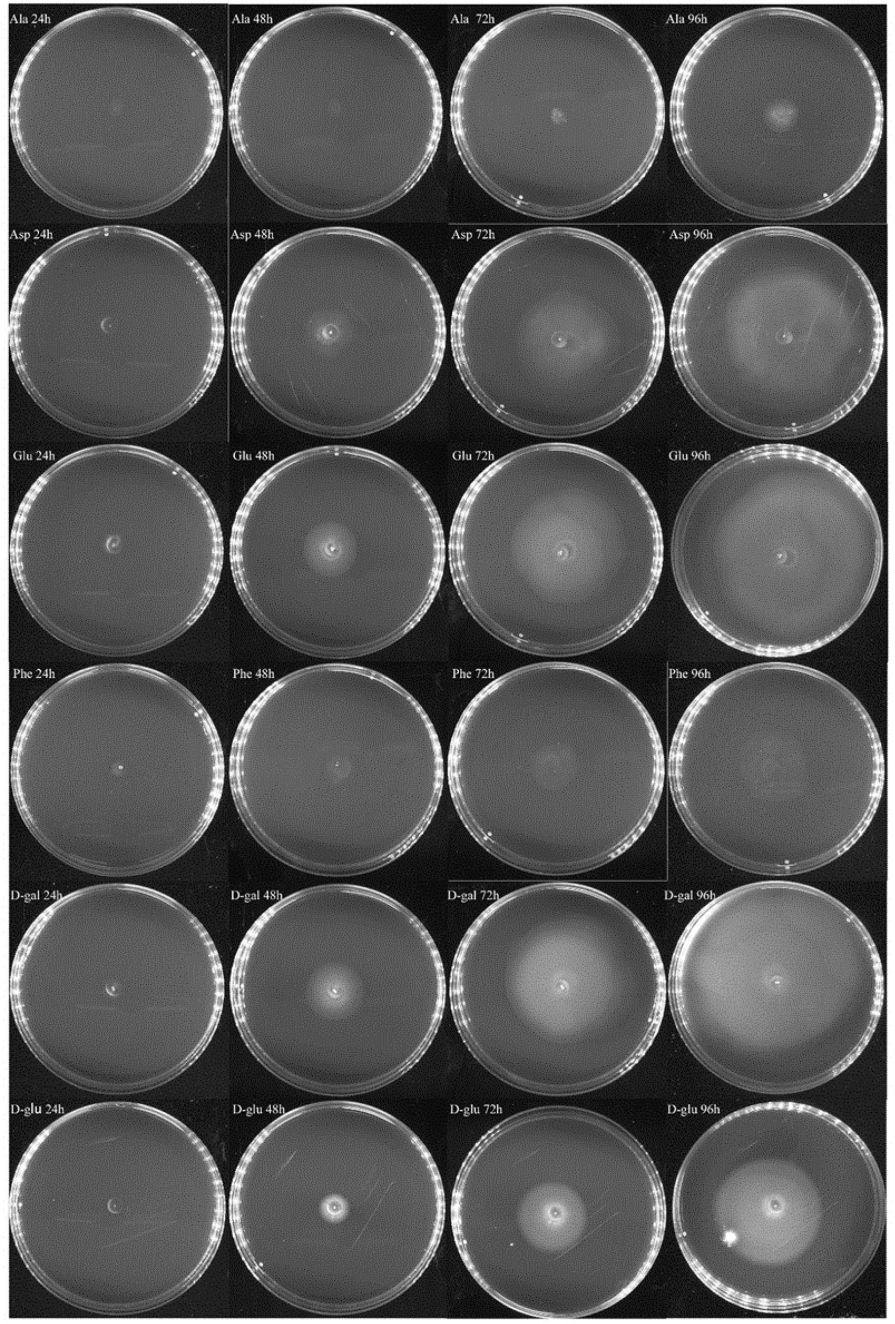
Chemotaxis of the regrown cells of *V. splendidus* persister cells. Each exogenous carbon source at a concentration of 400 mg·L^−1^ was separately added into M9 minimal plates containing 0.20% (w/v) agar; 1 ml M9 minimal medium was used to resuspend persister cells, and 10 μl bacterial suspension was dropped on M9 plate containing each carbon source, and the plates were incubated at 28°C for 96 h.

In the experiment to assess the chemotaxis ability of exponential cells and regrown cells of *V. splendidus* persister cells in the context of various carbon sources, three amino acids of L-glutamic acid, L-aspartic acid, and L-phenylalanine and two saccharides of D-galactose and D-glucose were chosen. L-alaine that could not revive the persister cells was selected to be used as a control. Ten microliters of each kind of cells was dropped on 0.2% M9 minimal plate containing each carbon source. We found that both exponential cells and regrown persister cells of *V. splendidus* showed obvious chemotaxis to 400 mg·l^−1^ L-glutamic acid, L-aspartic acid, L-phenylalanine, D-galactose, and D-glucose. After 96 h of treatment, the diameters of the *V. splendidus* on the plates containing L-alanine, L-aspartic acid, L-glutamic acid, L-phenylalanine, D-galactose, and D-glucose were, respectively, 3.13, 8.67, 8.65, 4.06, 9.93, and 8.74 cm, respectively (Figure 6), and the diameters of the regrown *V. splendidus* persister cells on the L-alanine, L-aspartic acid, L-glutamic acid, L-phenylalanine, D-galactose, and D-glucose plates were 1.93, 6.69, 8.15, 3.17, 8.57, and 5.48 cm, respectively (Figure 7). The chemotaxis of both exponential cells and regrown persister cells of *V. splendidus* showed the highest chemotaxis ability toward D-galactose and L-glutamic acid. On the contrary, the regrown cells of *V. splendidus* persister cells were not attracted by L-alanine within the 72 h.

### Exogenous Carbon Source Increased Tetracycline Susceptibility of *V. splendidus*

**Figure 8:**
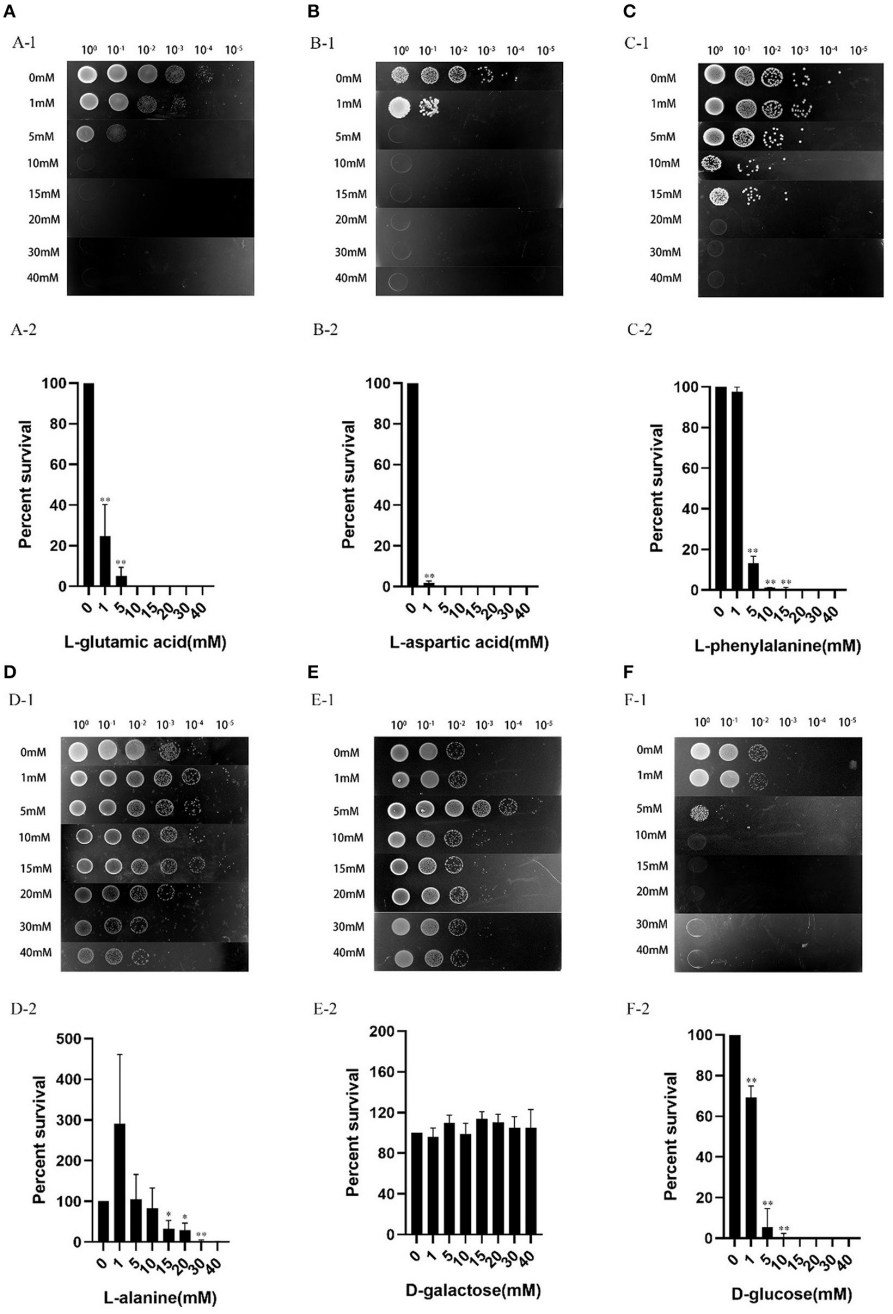
Effects of exogenous carbon sources on antibiotic susceptibility of *V. splendidus*. The overnight grown stationary phase culture was supplemented with the following concentrations of exogenous metabolites: **(A)** L-glutamic acid, **(B)** L-aspartic acid, **(C)** L-phenylalanine, **(D)** L-alanine, **(E)** D-galactose, and **(F)** D-glucose. The cultures without addition of any exogenous carbon sources were used as a control. The antibiotics and exogenous carbon sources were simultaneously added to the culture and incubated for 6 h, and the survived cells in different dilutions was represented by the colony formed on the plates (10^0^, 10^−1^, 10^−2^, 10^−3^, 10^−4^, and 10^−5^ meant no dilution, 10-, 100-, 1,000-, 10,000- and 100,000- dilutions). The relative percent of survival was calculated as follows: the cell number after tetracycline plus exogenous carbon source challenge/the cell number after tetracycline challenge alone. *p < 0.05 and **p < 0.01.

Studies have proved that the sensitivity of *E. coli* persister cells to aminoglycoside antibiotics is affected by exogenous glucose (23). In our present study, we tested whether the selected six metabolites, L-glutamic acid, L-aspartic acid, L-phenylalanine, L-alanine, D-galactose, and D-glucose could be used to increase the tetracycline susceptibility of *V. splendidus*. The results showed that the percentage of survived cells decreased with increased dose of L-glutamic acid, L-aspartic acid, L-phenylalanine, L-alanine, and D-glucose, with simultaneous addition of tetracycline (Figure 8). *V. splendidus* cells that were incubated with 400 μg·ml^−1^ tetracycline alone for 6 h were used as a control sample, and approximately 0.1%-1% cells survived after tetracycline treatment. Compared to *V. splendidus* grown in 400 μg·ml^−1^ tetracycline alone, the potion of the survived cells with the addition of carbon sources decreased significantly. The effect of exogenous carbon sources on the antibiotic sensitivity of *V. splendidus* was determined to be dose-dependent. The antibiotic sensitivity of *V. splendidus* also enhances when the concentrations rose from 0 to 40 mM. When the concentrations of L-aspartic acid, L-glutamic acid, D-glucose, and L-phenylalanine were 5, 10, 15, and 20 mM, *V. splendidus* cells were completely killed by 400 μg·ml^−1^ tetracycline; however, compared to *V. splendidus* in the control sample, the percentage of cell survival in the sample supplemented with 40 mM D-galactose showed no obvious change. These results suggested that the antibiotic susceptibility could be increased by addition of exogenous carbon sources.

## Discussion

Persister cells refer to a state of reduced metabolic activity that endows a subpopulation of isogenic bacteria with temporary multi-drug resistance, and they are able to revive the growth after the external stress is relieved. The development of persister cells can affect antibiotic efficacy, leading to incomplete treatment and repeated infections (35). Different kinds of bacterial persister cells have their preferred carbon source to wake up. The persister cells of *P. aeruginosa* revived in L-proline (17), while the persister cells of *E. coli* recovered in the presence of L-alanine (18). This study is the first attempt to explore the carbon sources that could wake up *V. splendidus* persister cells. *V. splendidus* persister cells can revive in the context of the amino acids including L-glutamic acid, L-aspartic acid, L-phenylalanine, L-leucine and L-arginine, and saccharides including D-galactose, maltose, mannose, sorbitol, D-fructose, N-acetyl-D-glucosamine, and D-glucose. For amino acids, L-glutamic acid revived *V. splendidus* persister cells used for the shortest time, and for saccharides, D-galactose, maltose, mannose, and sorbitol woke up *V. splendidus* persister cells better than D-fructose, N-acetyl-D-glucosamine, and D-glucose. The different numbers of colonies emerged on the specific carbon source probably attributed to the high heterogeneity of *V. splendidus* persister cells in dormancy (14), which resulted in their heterogeneous waking up on different carbon sources, probably due to different metabolism of various carbon sources in *V. splendidus*. Consequently, the results of this study indicated that (1) *V. splendidus* persister cells woke up responding to nutrient incentives similar to *P. aeruginosa* and *E. coli* (17, 18, 36) (2) L-glutamic acid is the best amino acid to revive *V. splendidus* persister cells, which is specific to this bacterial species; this further strengthened the concept that different bacterial persister cells preferred to use different carbon sources to wake up (17, 18).

Chemotaxis is the response to the stimulation of chemical substances in the external environment (37). Bacteria receive signals from external stimuli through chemoreceptors and process them through signal transduction systems to make corresponding movements (38). Chemotaxis plays an important role in the recovery of *E. coli* persister cells. Once persister cells resuscitated, the cells use chemotaxis to gain nutrients (18). In this study, we found that both exponential cells and persister cells of *V. splendidus* showed obvious chemotaxis to L-glutamic acid, L-aspartic acid, L-phenylalanine, D-galactose, and D-glucose; but relative to L-glutamic acid, L-aspartic acid, D-galactose, and D-glucose *V. splendidus* showed lower chemotaxis to L-phenylalanine, and L-alaine. The carbon sources that persister cells showed stronger chemotaxis were consist with the carbon sources that were suitable for the revival of persister cells, which suggested that the chemotaxis system may also play important roles in recognizing nutritional signals and resuscitation in *V. splendidus* persister cells, similarly to that in *E. coli* persister cells (18).

Further, we tested the effect of these exogenous carbon sources on the antibiotic susceptibility of *V. splendidus*. The addition of exogenous nutrients increases the antibiotic susceptibility of bacteria and has been reported in various pathogens. For example, glucose can alter cell metabolism, which leads to increased sensitivity to kanamycin and ciprofloxacin (22, 24) and aminoglycosides (21, 23), and this phenomenon is also found in *V. parahaemolyticu*s, *K. peneumoniae, S. aureus*, and *P. aeruginosa* (39–44). In the present study, we found that addition of exogenous carbon sources, L-glutamic acid, L-aspartic acid, L-phenylalanine, and D-glucose could also increase the tetracycline susceptibility of *V. splendidus* persister cells and this led us to speculate that these exogenous additions might trigger the little potion of persister cells at stationary phase to secede the persister state and enter into an active metabolic state. Compared with other methods to eliminate persister cells, such as development of new drugs, this method shows more effectiveness in combating antibiotic-resistant bacteria (24). Thus, we can surely believe that adding of the selected exogenous carbon sources combined with proper antibiotics will be a useful strategy to completely eliminate *V. splendidus*.

## Data Availability Statement

The original contributions presented in the study are included in the article/supplementary material, further inquiries can be directed to the corresponding authors.

## Author Contributions

GJ conducted the experiments, analyzed the data, and wrote the original manuscript. YanL conducted parts of the experiments and analyzed the data. YaL conducted parts of the experiments. WZ conceived and planned the research, supervised the research, revised the manuscript, and acquired funding. CL supervised the research and acquired funding. All authors reviewed the manuscript.

## Funding

This study was funded by the Zhejiang Provincial Natural Science Foundation for Distinguished Young Scholar (LR20C190001), the National Natural Science Foundation of China (31972833), the Zhejiang Provincial Natural Science Foundation (LZ19C190001), the Fundamental Research Funds for the Provincial Universities of Zhejiang (SJLZ2020001), and the K.C. Wong Magna Fund at Ningbo University.

## Conflict of Interest

The authors declare that the research was conducted in the absence of any commercial or financial relationships that could be construed as a potential conflict of interest.

## Publisher's Note

All claims expressed in this article are solely those of the authors and do not necessarily represent those of their affiliated organizations, or those of the publisher, the editors and the reviewers. Any product that may be evaluated in this article, or claim that may be made by its manufacturer, is not guaranteed or endorsed by the publisher.
